# High grade squamous intraepithelial lesion in inmates from Ohio: cervical screening and biopsy follow-up

**DOI:** 10.1186/1742-6413-3-15

**Published:** 2006-05-10

**Authors:** Daniela M Proca, Soraya Rofagha, Sedigheh Keyhani-Rofagha

**Affiliations:** 1 Department of Pathology, Ohio State University, Memorial Hospital of Union County, 500 London Ave, Marysville, OH 43040, USA; 2 Riverside Methodist Hospital, 3535 Olentangy River Road, Columbus, Ohio 43214, USA; 3 Department of Pathology, Ohio State University Medical Center, N-339 Doan Hall, 410 West 10^th ^Avenue, Columbus, Ohio 43210-1218, USA

## Abstract

**Background:**

Cervical carcinoma remains the second leading cause of cancer death in women worldwide and sexual behavior is regarded as the main contributing factor. We studied cervical cytology screening with surgical biopsy follow-up in women prisoners and compared the findings to those in the general population.

**Methods:**

We reviewed 1024 conventional cervical smears, 73 cervical biopsies and 2 loop electrosurgical excision procedure (LEEP) specimens referred to us from the Correctional Center in Columbus, Ohio during a 12-month period. The results were compared to 40,993 Pap smears from the general population for the same 12-month period.

**Results:**

High grade squamous intraepithelial lesion (HGSIL) was diagnosed in 1.3% of the cervical smears from the inmate population versus 0.6% in the general population (p < 0.01). The unsatisfactory rate was 1.6% compared to 0.3% in the general population (p < 0.01). Among the study population, follow-up tissue diagnosis was obtained in 24.3% of the abnormal cytology results (ASCUS, LGSIL, and HGSIL). Of the HGSIL Pap smears, 61.5% had a subsequent tissue diagnosis. Thirty-nine biopsies (52% of the all inmate biopsies and LEEP) showed CIN II/III (cervical intraepithelial neoplasia II/III). Eight of these thirty-nine follow-up biopsies diagnosed as CIN II/III had a previous cervical cytology diagnosis of ASCUS. The average age for HGSIL was 30.5 years (S.D. = 5.7) and for low grade squamous intraepithelial lesion (LGSIL) was 27.2 years (S.D. = 6.1).

**Conclusion:**

A significantly higher prevalence of HGSIL cervical cytology and unsatisfactory smears was encountered in female inmates, with tissue follow-up performed in less than two thirds of the patients with HGSIL. These results are in keeping with data available in the literature suggesting that the inmate population is high-risk and may be subject to less screening and tissue follow-up than the general population. Clinicians should proceed with urgency to improve screening and follow-up with treatment. The inmate population should be targeted for HPV vaccination promptly after FDA approval.

## Background

Cervical carcinoma remains the second leading cause of cancer death in women worldwide. Risk factors for cervical squamous carcinoma include early onset of sexual activity, early age of first pregnancy, multiple sexual partners, exposure to human papilloma virus (HPV), cigarette smoking, immunosuppression, human immunodeficiency virus or concomitant neoplasia of vulva or vagina. [1-3] Adequacy and frequency of cytological screening may also play an important role, the risk of developing invasive squamous cell carcinoma in women who had no Papanicolaou smear in the last five years being 3.7–4.3 times greater than in women who had one in the past two years.[1] The risk of death from invasive cervical carcinoma is highest in women with inadequate cytological screening.

Women inmates represent a socially disadvantaged group with high-risk behavior and low participation in the Pap smear screening programs, therefore, they have a higher propensity for developing cervical carcinoma than the general population. [4-8] Prevalence rates as high as 10% for carcinoma in situ, 16% for syphilis, and 5% for gonorrhea have been reported in detainees.[4,5] We studied the results of cervical screening in female prisoners, in addition to cytology specimen adequacy, and biopsy/loop electrosurgical procedure specimens (LEEP) follow-up.

## Methods

We reviewed the cytology reports for 1024 cervical conventional smears performed and referred to us from the Correctional Center in Columbus, Ohio during a 12-month period. Our laboratory was the exclusive referral laboratory for this institution. The Papanicolaou-stained conventional cervico-vaginal smears were classified according to the Bethesda system (1988): within normal limits (WNL), atypical squamous cells of uncertain significance (ASCUS), atypical glandular cells of unknown significance (AGUS), low grade squamous intraepithelial lesion (LGSIL), high grade squamous intraepithelial lesion (HGSIL), carcinoma, and unsatisfactory for evaluation.[9] The prevalence of abnormal cytological results and unsatisfactory smears in the inmate population was compared to the general population represented by 40,993 conventional Pap smears referred to us for screening over the same 12 month-period. A "z" test for comparing proportions was used to assess statistical significance.

We also studied the age distribution of HGSIL and LGSIL in the prisoner population. The total number of follow-up biopsies/LEEP over the same time period was 75 and these were performed in inmates who had cytological diagnoses of ASCUS, LGSIL, and HGSIL. This research was conducted with approval of the Institutional Review Board at The Ohio State University (Protocol number 2002H0089).

## Results

HGSIL was diagnosed in 1.3% of the cervical smears from the inmate population versus 0.6% in the general population (p < 0.01). LGSIL was diagnosed in 5.9% versus 5.7%, respectively. Atypical smears (ASCUS/AGUS) were seen in 9.4% of cases within the inmate population versus 10.2% within the general population. The unsatisfactory rate was 1.6% compared to 0.3% in the general population (p < 0.01) (Table 1). Smears were unsatisfactory for cytological evaluation due to insufficient material (50%), heavy inflammation (31.3%) and obscuring blood (18.7%).

**Table 1:** Number and percent of cytological diagnoses in the general versus inmate population

Population	Total	WNL	Atypia	LGSIL	HGSIL	Carcinoma	Unsatisfactory
General (%)	40933 (100%)	34066 (83.1%)	4181 (10.2%)	2337 (5.7%)	246 (0.6%)*	41 (0.1%)	122 (0.3%)*
Inmate (%)	1024 (100%)	839 (81.9%)	96 (9.4%)	60 (5.9%)	13 (1.3%)*	0 (0%)	16 (1.6%)*

*p < 0.01

The age distribution of Pap smear abnormalities in the study population was studied. The average age for HGSIL was 30.5 years (S.D. = 5.7), while that for LGSIL was 27.2 years (S.D. = 6.1).

A total of 73 biopsies and 2 LEEP were performed at the Correctional Center and sent to us for evaluation within the same 12 month-period. These specimens provided the tissue follow-up of abnormal cytologic results. Follow-up tissue diagnosis was obtained in 24.3% of the abnormal cytology results (ASCUS, LGSIL, and HGSIL). Of the HGSIL Pap smears, only 61.5% had a subsequent tissue diagnosis. Thirty-nine biopsies (52% of the all inmate biopsies and LEEP) showed CIN II/III (cervical intraepithelial neoplasia II/III). Eight of these thirty-nine follow-up biopsies diagnosed as CIN II/III had a previous cervical cytology diagnosis of ASCUS. On re-evaluation, these missed HGSIL cervical smears showed obscuring blood, heavy inflammation, thick areas, drying artifact and/or poor fixation, many being regarded as inadequate for diagnosis.

## Discussion

Cervical squamous intraepithelial lesions are more common in teenagers, HIV positive women and socially disadvantaged populations, [10-15] findings shown to be related not only to the last few decades' sexual revolution, but also to deficient Pap smear screening.[10,12] While the prevalence of preneoplastic lesions has been rising, the age at diagnosis has been falling, reaching a peak between ages 20 and 30[2,3,11,13].

Female inmates represent a population at risk of developing invasive cervical cancer, because of their high-risk sexual behavior, their low participation in cytological screening programs and the difficulty of consistent follow-up of abnormal results. [4-8] We report the results of routine cervical screening in detainees over a 12 month-period, as well as histological follow-up of abnormal cervical cytology in this same patient population.

Similar to previous studies, we found that detainees have a higher prevalence of HGSIL cervical cytology and have less tissue follow-up than the general population.[4,6-8] Overall, tissue follow-up was performed in less than one-fourth of inmates with an abnormal cytology result and less than two-thirds of inmates with a cytology result of HGSIL.

Furthermore, significantly higher numbers of unsatisfactory and suboptimal smears were encountered, when compared to the general population (1.6% versus 0.3%). Eight of the thirty-nine biopsies interpreted as CIN II/III (20.5%) had a previous diagnosis of ASCUS on cervical cytology. All Pap smears (from the inmates and from the general population) were conventional. Therefore, the difference in unsatisfactory smears cannot be explained by differences between conventional and liquid based cytology. Pap smears from the inmate population were found inadequate due to two main causes: poor collection (insufficient material, air drying artifact and thick smears) and coexistent cervical pathology (bleeding, heavy inflammation).

The average age at diagnosis for HGSIL and LGSIL in our patient population was similar to that previously reported for the general population.[2,3,11] Different from other reports is that our population of detainees had more severe abnormalities on Pap smear at an earlier age[4,6,7].

## Conclusion

Our study revealed that women prisoners have higher rates of unsatisfactory cervical cytology smears, as well as an increased prevalence of cervical HGSIL. The increased prevalence of HGSIL in this population may be due to undetected lesions due to suboptimal specimens or inadequate population screening. Suboptimal specimens were found to be due to poor collection (insufficient material, air drying artifact or thick smears) or coexistent cervical pathology (infection, bleeding or heavy inflammation). The increased prevalence of HGSIL may also be due to inadequate or limited health resources in this population, which may contribute to the lack of consistent and regular screening. Furthermore, less than two-thirds of inmates with HGSIL cytology had a follow-up tissue diagnosis. This data reinforces the need for clinicians to proceed with systematic screening and consistent follow-up and treatment of this high-risk population with urgency, especially because of the more transient nature of the inmate population. Further epidemiologic studies relating risk factors, including demographic characteristics, sexual health behavior and health care delivery, to morphologic findings are needed to help clarify the causal relationships between the increased prevalence of HGSIL and our study population. Nonetheless, the higher prevalence of HGSIL in combination with the unsatisfactory rate of smears likely contributes to the higher rates of invasive squamous cell carcinoma of the cervix reported in the literature for this population and helps identify these women as a high-risk group. Female inmates should be targeted with preventive and therapeutic measures to eliminate this health disparity. In addition to efforts to improve screening and follow-up, female inmates should be a target population for immunization with the HPV vaccine promptly after FDA approval.

## Abbreviations

loop electrosurgical excision procedure (LEEP)

atypical squamous cells of unknown significance (ASCUS)

atypical glandular cells of unknown significance (AGUS)

within normal limits (WNL)

high grade squamous intraepithelial lesion (HGSIL)

low grade squamous intraepithelial lesion (LGSIL)

human papilloma virus (HPV)

cervical intraepithelial lesion (CIN)

## Competing interests

The author(s) declare that they have no competing interests.

## Authors' contributions

DP performed the retrospective review, performed the literature search and prepared the manuscript. SR assisted writing the manuscript and helped with statistical analysis. SKR designed the study and supervised and revised the manuscript.
